# Yttrium Residues in MWCNT Enable Assessment of MWCNT Removal during Wastewater Treatment

**DOI:** 10.3390/nano9050670

**Published:** 2019-05-01

**Authors:** Justin Kidd, Yuqiang Bi, David Hanigan, Pierre Herckes, Paul Westerhoff

**Affiliations:** 1 Nanosystems Engineering Research Center for Nanotechnology Enabled Water Treatment, School of Sustainable Engineering and The Built Environment, Arizona State University, Tempe, AZ 85287-3005, USA; justin.kidd@asu.edu (J.K.); yuqiangb@asu.edu (Y.B.); 2 Department of Civil and Environmental Engineering, University of Nevada, Reno, NV 89557-0258, USA; dhanigan@unr.edu; 3 School of Molecular Sciences, Arizona State University, Tempe, AZ 85287-1604, USA; pierre.herckes@asu.edu

**Keywords:** MWCNTs, wastewater treatment, yttrium, RAS, spICP-MS, ICP-MS

## Abstract

Many analytical techniques have limited sensitivity to quantify multi-walled carbon nanotubes (MWCNTs) at environmentally relevant exposure concentrations in wastewaters. We found that trace metals (e.g., Y, Co, Fe) used in MWCNT synthesis correlated with MWCNT concentrations. Because of low background yttrium (Y) concentrations in wastewater, Y was used to track MWCNT removal by wastewater biomass. Transmission electron microscopy (TEM) imaging and dissolution studies indicated that the residual trace metals were strongly embedded within the MWCNTs. For our specific MWCNT, Y concentration in MWCNTs was 76 µg g^−1^, and single particle mode inductively coupled plasma mass spectrometry (spICP-MS) was shown viable to detect Y-associated MWCNTs. The detection limit of the specific MWCNTs was 0.82 µg L^−1^ using Y as a surrogate, compared with >100 µg L^−1^ for other techniques applied for MWCNT quantification in wastewater biomass. MWCNT removal at wastewater treatment plants (WWTPs) was assessed by dosing MWCNTs (100 µg L^−1^) in water containing a range of biomass concentrations obtained from wastewater return activated sludge (RAS) collected from a local WWTP. Using high volume to surface area reactors (to limit artifacts of MWCNT loss due to adsorption to vessel walls) and adding 5 g L^−1^ of total suspended solids (TSS) of RAS (3-h mixing) reduced the MWCNT concentrations from 100 µg L^−1^ to 2 µg L^−1^. The results provide an environmentally relevant insight into the fate of MWCNTs across their end of life cycle and aid in regulatory permits that require estimates of engineered nanomaterial removal at WWTPs upon accidental release into sewers from manufacturing facilities.

## 1. Introduction

Multi-walled carbon nanotubes (MWCNTs) consist of multiple rolled layers (concentric tubes) of graphene, which bring rise to their unique material properties (e.g., high thermal conductivity, elasticity, tensile strength, microwave absorbency, etc.). MWCNTs are being widely considered in numerous applications such as nanosensors [1], nanocomposite materials [2], paint or other coatings [3], and field-emission displays [4]. Manufacturing processes often combine MWCNTs into stock solutions containing surfactants to maintain their dispersions or into polymers, which prevents working with dry MWCNT powders. Accidental MWCNT spills at manufacturing facilities can result in discharges into sewers, which flow to local wastewater treatment plants (WWTPs). In direct spillage scenarios into rivers or lakes, predicted MWCNT concentration ranges in surface waters may be in the microgram per liter (µg L^−1^) range [5,6], which is 10–1000 times lower than MWCNT levels estimated to cause adverse acute biological responses in aquatic organisms [7,8,9]. However, in situations where MWCNT spills occur within industrial facilities, a short duration influx of MWCNTs at higher concentrations can occur at wastewater treatment plants (WWTPs). If not removed at WWTPs, short duration releases of MWCNTs into receiving waters could occur. Evaluating MWCNT removal in bench scale conditions can approximate full scale operations and provide useful information on what might be expected at full scale [10]. While research has shown that WWTPs can remove >90% of many types of nanoparticles due to their association with wastewater-activated sludge and its subsequent physical separation [11,12,13], the industry needs validated methods to estimate nanoparticle removal at WWTPs, similar to strategies employed for chemical pollutant removal tests (e.g., OPPTS 835.1110 EPA 712-C-98-298).

Current analytical techniques to detect and quantify carbon nanoparticles include programmed thermal analysis (PTA), Raman spectroscopy, UV-visible spectrophotometry, and microwave treatment with thermal analysis, but most available techniques have detection limits in solid matrices and water on the order of 10–100 mg kg^−1^ or 0.1–10 mg L^−1^, respectively [14]. Recent studies evaluated the ability of single particle inductively coupled plasma-mass spectrometry (spICP-MS) to detect residual trace catalytic metals (e.g., Fe, Y, Ni, Co, Mo) that persisted in single- or multi-walled carbon nanotubes (SWCNTs) at ng L^−1^ levels in an aqueous matrix [15,16,17,18,19,20,21,22,23,24]. Therefore, we explored spICP-MS and ICP-MS directly for the detection of known specific commercial MWCNTs in complex wastewater matrices. Selection of a trace-element to use spICP-MS in this application must meet the following criteria: (1) be present in the MWCNT of interest, (2) not dissolve upon contact of the MWCNT with water, and (3) have low background occurrence in common municipal wastewater.

The goal of this research was to demonstrate the use of metals as a surrogate measurement for MWCNT concentrations and use the measurements to assess the fate of MWCNTs at WWTPs under realistic MWCNT sewage concentrations. Specific objectives included (1) to select a trace rare earth element (REE) residual in MWCNTs as a surrogate for MWCNT quantification in wastewater by ICP-MS, (2) to determine the association of the selected REE (Y) and MWCNTs using transmission electron microscopy (TEM) before and after dissolution studies, (3) to determine the removal of MWCNTs with wastewater return activated sludge (RAS), and (4) to use MWCNT removal results to predict conditions following an industrial spill scenario.

## 2. Materials and Methods

### 2.1. Preparation of MWCNT Solutions

A MWCNT working solution was prepared by diluting a commercial 5% MWCNT solution (Altana, Wesel, Germany) to approximately 200 mg L^−1^ using 18.2 MΩ-cm Nanopure water in 50 mL polypropylene centrifuge tubes. The MWCNT working solution was hand shaken and then immersed in a batch sonicator (Branson 5800, Branson Ultrasonics, Danbury, CT, USA) to disperse MWCNT particles. A test solution was prepared following prior research for wastewater biomass-nanoparticle removal evaluation [25], using 1 mM NaHCO_3_ (pH 7.4) prepared in Nanopure water (≥18.2 MΩ-cm). Four vial types were used in these experiments: (1) 50 mL polystyrene centrifuge vials, (2) 40 mL borosilicate vials with a septa screw cap, (3) 40 mL amber glass vials with a Teflon septa screw cap, and (4) a standard jar test apparatus (Phipps and Bird, Richmond, VA, USA) with six 2 L polyethylene vessels.

### 2.2. Return Activated Sludge (RAS) Stock Preparation

RAS was collected in 1 L Nalgene HDPE wide mouth bottles from a metro-Phoenix region secondary WWTP practicing nitrification and denitrification. The same collection approach was used previously for nanoparticle–WWTP studies [10,11,25]. Immediately after collection, the sample bottles were placed in an ice cooler (approx. 4 °C) and returned to the lab where they were stored in a 4 °C refrigerator. Within 24 h, the solids visually settled, and the wastewater supernatant was decanted. The RAS was re-suspended in 400 mL of 1 mM NaHCO_3_ solution, allowed to settle, and the supernatant discarded again. This rinsing procedure was repeated for a total of three times. A final RAS stock suspension was then made in a 1 L Nalgene HDPE bottle with 1 mM NaHCO_3_ solution. To determine the concentration of the biomass suspension (g TSS (total suspended solids) L^−1^), the collected biomass was stirred with a stir bar to shear larger particles and obtain a more uniform particle size. 10 mL of the biomass sample was vacuum filtered with a 0.45-micron glass fiber filter, followed by 3 rinses of the filter using 10 mL of Nanopure water. The filter with biomass was then carefully removed, placed into an aluminum weighing dish, and dried at 105 °C until the change in biomass weight between sampling periods (10 min) was less than 4% of total sample weight. The weight of the dry biomass was recorded, and the concentration of dry biomass was calculated (g TSS L^−1^). The stock RAS suspension was then diluted with 1 mM NaHCO_3_ solution to achieve a TSS concentration equal to the desired TSS concentration in the experimental samples. New diluted stocks were made from the original stock for each experimental RAS concentration. The new RAS stocks were stored in the 4 °C fridge until the experiment was conducted. We completed the experiment within 24 h of rinsing and diluting the RAS stock solutions.

### 2.3. Quantification Techniques for MWCNTs

#### 2.3.1. Light Scattering Detection and Programmed Thermal Analysis of MWCNTs

To supplement the use of trace elements for MWCNT detection, MWCNTs were also quantified using UV-Vis spectroscopy (Hach DR5000, Loveland, CO, USA) and PTA [26]. For UV-Vis spectroscopy, calibration curves were made by creating a dilution series from the 200 mg L^−1^ MWCNT stock solution in a 1 mM NaHCO_3_ matrix solution. The dilution series was scanned at wavelengths from 300 to 800 nm (Figure S1). For PTA, the 5% MWCNT solution and a MWCNT powder were analyzed. Both samples used the same manufactured MWCNTs. The MWCNT solution was dried at 105 °C until the change in dry weight measured between 24-h time points was <4%. The two MWCNTs were placed in a furnace and heated in the absence and then presence of oxygen following the methodology outlined previously for PTA detection of MWCNTs (Figure S2) [26,27,28,29].

#### 2.3.2. Microwave Digestion of MWCNTs

Soluble metal residuals present on MWCNTs were quantified by ICP-MS after microwave digestion. Briefly, ~0.1 g of 5% MWCNT solutions were digested in 10 mL HNO_3_ (70%) with a CEM MARS 5 microwave accelerated reaction system (1200 watts, 2450 MHz; CEM Matthews, NC, USA) at 170 °C for 40 min. After cooling, the digested samples were diluted in 2% HNO_3_ and analyzed for 30 common metals using a X-Series II ICP-MS (Thermo Scientific, Waltham, MA, USA). A previous study indicated that this method yielded reliable results for the quantification of metal contents in CNTs [30]. Of the 54 elements scanned, only a few elements were found in measurable concentrations in this product.

#### 2.3.3. Dissolution Tests and TEM Imaging to Assess Residual Metal Association with MWCNTs

To determine association of metal residuals with the MWCNTs, two 40 mL solutions of MWCNTs (1 mg L^−1^) were suspended in 50 mL polystyrene centrifuge vials at different pH values: 2% HNO_3_ (pH 2) and 1 mM NaHCO_3_ (pH 7.4). Solutions were shaken for 24 h to evaluate the release of metal catalysts. 15 mL of each solution passed through a 30 kDa ultrafilter using a centrifuge for 15 min at 5000 G (Millipore, Ultracel Regenerated Cellulose Membrane, >90% recovery, Burlington, MA, USA) to separate remaining MWCNT particulates and nano-scale or dissolved metals. The permeate was analyzed by spICP-MS. The remaining 25 mL of MWCNT solutions not used in the ultrafiltration process was evaluated with transmission electron microscopy (TEM) coupled with energy dispersive X-ray spectroscopy (EDAX) (Philips CM200, Amsterdam, Netherlands). Samples were placed on copper TEM grids (2% HNO_3_ sample was neutralized with NaOH salt before placement on grids) and analyzed.

#### 2.3.4. Single Particle ICP-MS and ICP-MS Analysis of Yttrium

We operated ICP-MS in single particle mode following methods published previously [23,31,32,33] and followed the approach of analyzing for trace metals in MWCNTs as demonstrated for SWCNTs [15,23]. For samples analyzed with spICP-MS, if the sample contains dissolved metals, the ions will be distributed homogenously within the solution, producing a consistent intensity signal vs. time across readings. However, if the sample contains particles, the metal atoms within the sample are no longer distributed homogenously. Instead, the metals are present as discrete particulates and, once ionized, move through the mass analyzer to the detector as a cluster of ions. This cluster of ions results in a spike above the background, where the pulse corresponds to an individual particle and the background represents the “dissolved” metals in solution.

Of the six metal residuals (^59^Co, ^60^Ni, ^68^Zn, ^89^Y, ^90^Zr, and ^95^Mo) that were physically bound to the MWCNTs, we determined that yttrium (Y) would be best used as an indicator of this specific MWCNT for quantification because it has the highest sensitivity on ICP-MS (see the Results section) and also a low background concentration of Y is present in wastewater sludge [34]. Analysis by spICP-MS was performed on non-digested samples using the X-Series II ICP-MS (Waltham, MA, USA) in time-resolved data acquisition mode with a dwell time of 10 ms following methodologies described elsewhere [35]. In brief, samples were placed in polypropylene sample tubes in Nanopure water, and the tubes were placed in a sonicating bath for 15 min. Samples were then immediately pumped into the instrument, and the spectra for ^89^Y were recorded. We used three times the standard deviation of the spectra to delineate MWCNT pulses from background dissolved metal. We also measured total dissolved ^89^Y concentrations in MWCNT suspension by ICP-MS by diluting the MWCNT stock solution to between 0.001 to 100 µg L^−1^. A linear calibration curve was made between dissolved Y concentrations and Y responses (in counts per second, cps) (Figure S3). Then we calculated a minimum detection level (MDL) following the United States Environmental Protection Agency (EPA) procedure of running 10 replicate samples. The MWCNT stock solution was diluted to roughly 2 µg L^−1^ to determine the MDL (0.49 µg L^−1^). Because we found that the Y was easy to dissolve, biomass partitioning experiments were analyzed by first exposing the biomass/CNT mixture to 2% nitric acid, followed by filtration through 30 kDa ultrafilters and ICP-MS analysis. Experimental samples were diluted to between 0.1 and 2 µg L^−1^ and analyzed on ICP-MS. A linear calibration curve was made between MWCNT concentrations and Y responses (in counts per second, cps) (Figure S4).

### 2.4. MWCNT-RAS Batch Interaction Experiments

For experiments conducted in 40 mL glass and amber vials with septa screw caps, triplicate samples with 35 mL of water were dosed with clean wastewater biomass to achieve the desired final biomass concentrations of 0, 0.5, 1.0, 2.0, 3.0, and 5.0 g TSS L^−1^. The MWCNT solution (5 mL) was added to a final MWCNT concentration of 100 µg L^−1^ for a total reactor volume of 40 mL. Triplicate controls with wastewater biomass only and nanoparticle solution only were also performed. Vials were sealed and secured on a rotator table for 3 h at 45 revolutions per minute (rpm). After mixing, the samples equilibrated in the lab for 1 h to allow for biomass settling, and a 10 mL supernatant aliquot was taken and centrifuged at 150 G for 10 min to settle any remaining biofloc in the supernatant. The samples were transferred to clean vials and acidified with 2% HNO_3_ for 24 h to release Y from the MWCNTs. Samples were passed through 30 kDa ultrafilters and analyzed using ICP-MS.

For jar test experiments, a standard jar test apparatus (Phipps and Bird, Richmond, VA, USA) was used with six 2 L vessels. Each jar was given a different biomass concentration (0, 0.5, 1.0, 2.0, 3.0, and 5.0 g TSS L^−1^), and these biomass concentrations were made up to 1.8 L in a 1 mM NaHCO_3_ matrix solution. The no biomass control contained 1.8 L of 1 mM NaHCO_3_ matrix solution. The experiments were run in triplicate (*n* = 18). After biomass was added at specified concentrations, 200 mL of 1 mg L^−1^ MWCNT solution was added to each reactor to give a final MWCNT concentration of 100 µg L^−1^ and a final reactor volume of 2 L. The jar apparatus mixing was continuous at 45 rpm for 3 h. The apparatus was turned off, and the biomass settled for 1 h. 50 mL of supernatant from each jar test reactor was placed into a 50 mL polystyrene centrifuge vial and centrifuged at 150 G to separate the remaining biofloc from the solution. The supernatants were then acidified with 2% HNO_3_ for 24 h and filtered through 30 kDa ultrafilters prior to ICP-MS analysis of dissolved Y concentrations. Randomly chosen samples were also analyzed for ^89^Y using spICP-MS to confirm removal of pulses originating from Y in the MWCNTs.

## 3. Results and Discussion

### 3.1. Characterization of MWCNTs and the Trace Metal Residuals Associated with MWCNTs

**Figure 1:**
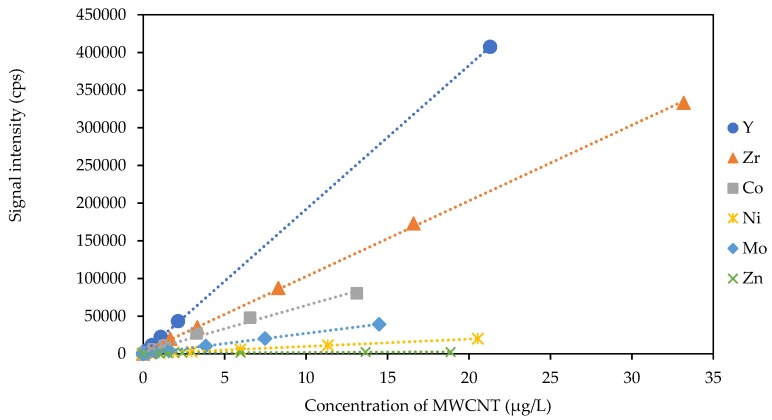
Representative linear sensitivity of trace metal residuals (Y, Zr, Co, Ni, Mo, Zn) using ICP-MS for a range of MWCNT concentrations dosed into water. The ratio of net signal to concentration is the sensitivity of each element (see Table 1).

Elemental content of the MWCNTs determined by ICP-MS after microwave digestion are summarized in Table 1. Of the 54 elements scanned, only a few elements were found in measurable concentrations in this product. Quantitative analyses were conducted for ^59^Co, ^60^Ni, ^68^Zn, ^89^Y, ^90^Zr, and ^95^Mo (Table 1). Data are shown in Figure 1 for these elements; in all other calibration curves for this research, signal intensity responses were linear, fitting the data with high R^2^ across concentration ranges from non-detectable in blanks without MWCNT to over 1 ppm MWCNT.

Iron (Fe) was found at the highest concentration (~9.36 mg g^−1^) followed by Zr (~1.25 mg g^−1^), Co (153 μg g^−1^), Y (76.2 μg g^−1^), Zn (10.5 μg g^−1^), Ni (5.1 μg g^−1^), and Mo (1.2 μg g^−1^). We presume these residuals are catalyst impurities in the metals used in MWCNT synthesis rather than intentionally added by manufacturers. These results align well with reports of Y and Co in SWCNTs [15,17] Figure 1 shows the sensitivity of these three metal residuals by ICP-MS after microwave digestion of the CNT.

Sensitivity was found by comparing the ratio of their net signal (counts per second) to concentration (μg L^−1^) and is important because the quantitation limit of elements by ICP-MS is primarily set by sensitivity. We chose to normalize instrument response to the MWCNT concentration rather than to a neat metal standard because the manufacturer MWCNT solution matrix is confidential, causing unknown ionization or instrumental effects that would not be present for a neat solution. The selection of Y was based upon multiple criteria. First, Y had the highest sensitivity of the three metal residuals, followed by zirconium and cobalt. Second, Y did not dissolve out of the MWCNT and into water upon addition (discussed in detail below). Third, we previously reported that Y had the lowest *enrichment factor* (EF), indicating few anthropogenic sources, and occurred at very low to non-detectable concentrations in wastewater or wastewater return activated sludge [26]. As a result, we determined that Y was a feasible residual metal candidate to quantify MWCNT concentrations using spICP-MS and ICP-MS.

**Figure 2:**
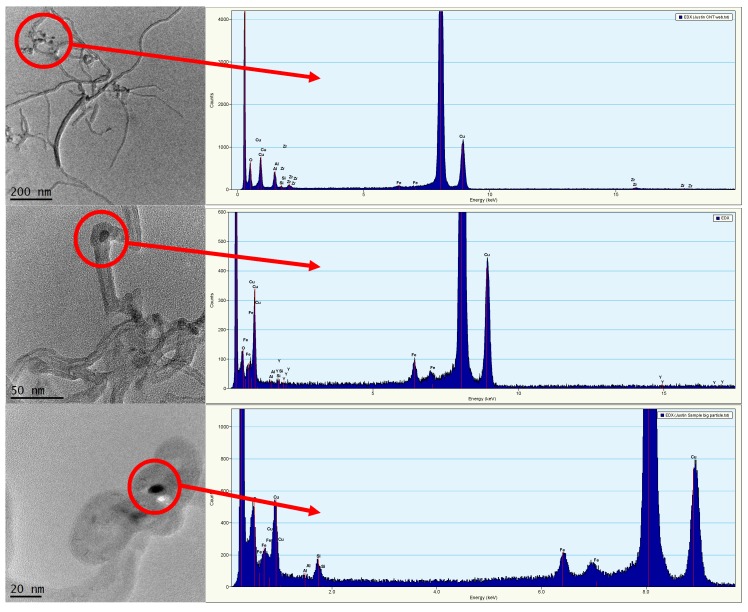
Transmission electron microscopy (TEM) imaging of 5% MWCNT stock solution and corresponding energy dispersive X-ray spectroscopy (EDAX) of highlighted TEM images. Dense (darker) regions on MWCNTs are residual trace metals remaining after MWCNT synthesis.

TEM images and their corresponding EDAX for the 5% MWCNT solution are shown in Figure 2. The TEM images are representative of analytical replicates taken from the stock solution. The average width of the MWCNTs was found to be 18 ± 3 nm, and the length was between 50 and 5000 nm. Additional TEM images are in Figure S5. EDAX detected zirconium (Zr), aluminum (Al), and oxygen (O) with lower levels of phosphorus (P), iron (Fe), and silica (Si) present in the dense regions embedded within the MWCNTs. Trace metals quantified by ICP-MS were not detected by EDAX, which often is only sensitive at >0.5% weight.

### 3.2. Association of Yttrium with MWCNTs

Dissolution experiments using MWCNTs were performed to determine if Y was mobilized into water or retained on the MWCNTs. In control experiments, we expected that dissolved Y would pass through the 30 kDa ultrafilters; however, if Y remained associated with the MWCNTs it would be retained by the 30 kDa ultrafilters. All of the ^89^Y standard passed through the ultrafilter, confirming that dissolved Y is not retained or sorbed by the ultrafilter (>90% recovery).

**Figure 3:**
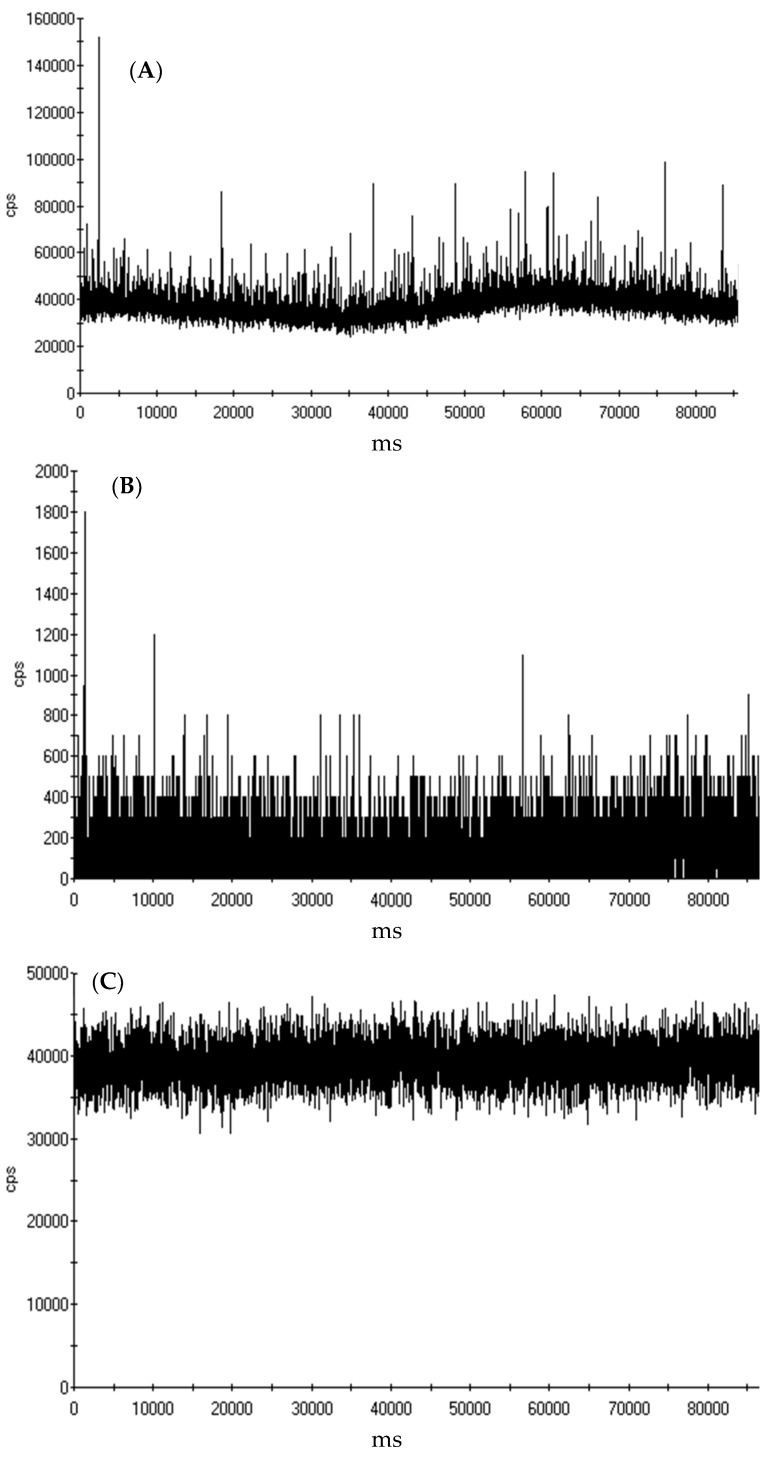
Yttrium single particle mode inductively coupled plasma mass spectrometry (spICP-MS) data of an (**A**) undissolved initial MWCNT sample, (**B**) undissolved MWCNT permeate, and (**C**) 2% HNO_3_ dissolved MWCNT permeate passed through a 30 kDa ultrafilter, showing that Y did not pass through the ultrafilter unless acidified and dissolved by 2% HNO_3_.

To determine the initial Y pulses present, 15 mL of the MWCNT solution was analyzed with spICP-MS (Figure 3A). A large number of pulses were present, and the signal (y-axis) baseline was between 40,000 and 60,000 cps for the Y response. After filtering 15 mL of the MWCNT solution through a 30 kDa ultrafilter (Figure 3B), there were virtually no pulses, and a baseline of <1000 cps. When the remaining 15 mL of the MWCNT solution was acidified in 2% HNO_3_ for 24 h and filtered through the 30 kDa filters (Figure 3C), there were no pulses present, but the baseline shifted from <1000 cps to approximately 40,000 cps. Taken together, we concluded that in 1 mM NaHCO_3_, Y was physically bound with MWCNTs and did not pass through the filter into the permeate. Once the MWCNTs were acidified with 2% HNO_3_, the Y dissolved and passed through the ultrafilter, further indicating that Y was associated with the MWCNTs.

**Figure 4:**
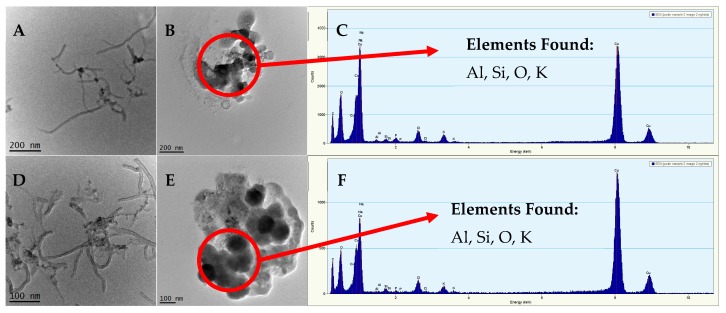
(**A**) TEM image of MWCNT in 1 mM NaHCO_3_ matrix solution after initial spike (<1 h), (**B**) TEM image of MWCNT in 2% HNO_3_ matrix solution after initial spike (<1 h), (**C**) EDAX of MWCNT from image B, (**D**) TEM image of MWCNT in 1 mM NaHCO_3_ matrix solution after 24 h, (**E**) TEM image of MWCNT in 2% HNO_3_ matrix solution after 24 h, (**F**) EDAX of MWCNT from Image E.

To determine if Y leached from the MWCNTs over time, MWCNTs were spiked into either 1 mM NaHCO_3_ or 2% HNO_3_ solutions, and TEM/EDAX was conducted at the time points of 0 h and 24 h (Figure 4). Figure 4A–C shows TEM and EDAX images of MWCNTs in 1 mM NaHCO_3_ and 2% HNO_3_ after the initial spike of MWCNTs into the solution. Figure 4D–F shows TEM and EDAX images of MWCNTs in 1 mM NaHCO_3_ and 2% HNO_3_ after 24 h. In the 1 mM NaHCO_3_ solution, MWCNTs still contained residual metals (Fe, Al, etc.) after 24 h, and there was no change in MWCNT morphology. In the 2% HNO_3_ solution, no MWCNTs remained at either time point (0 h and 24 h). Instead, we observed bundles of metal catalysts in the solution. We concluded that the residual metals were tightly associated with the MWCNTs in 1 mM NaHCO_3_ and were not likely to leach into the solution during our wastewater activated sludge experiments under 24 h. We also concluded that the low pH of the 2% HNO_3_ solution caused metal dissolution from the MWCNTs.

### 3.3. Quantification of Yttrium in MWCNT by spICP-MS and ICP-MS

**Figure 5:**
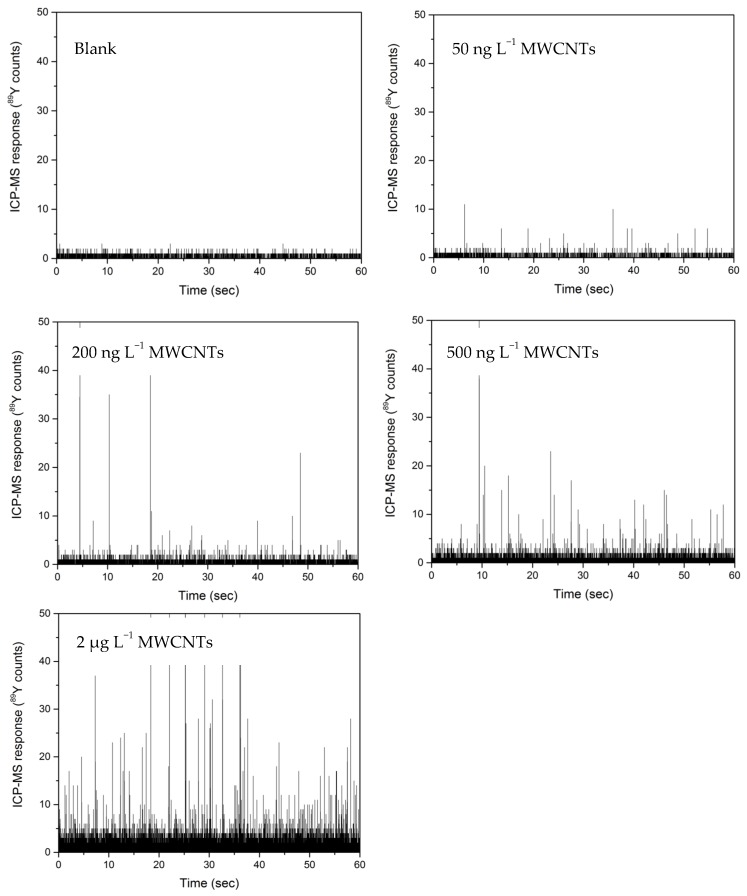
Single particle ICP-MS detection using ^89^Y of MWCNTs in a solution across a range of concentrations plus a control (blank) sample.

Our investigation used spICP-MS to quantify Y in MWCNTs. ^89^Y showed a linear relationship for MWCNT solutions between 50 ng L^−1^ and 20 µg L^−1^ (Figure 5). Increasing MWCNT concentration led to more pulses, with Y pulses used as a measurement of MWCNT concentration. The total pulse intensity within a specified run time was integrated by the number of pulses and used to correlate with MWCNT concentrations using the same approach for SWCNTs [15,17]. Following the EPA method 200.8, an MDL for MWCNTs was calculated to be 0.82 µg L^−1^ (Figure S6).

Separately we determined if dissolved Y, after acidification, could be quantified by ICP-MS instead of using spICP-MS because conventional ICP-MS is more commonly available in industry and commercial analytical labs. The 5% MWCNT solution was diluted in ultrapure water to 2 µg L^−1^, and a dilution series was made and acidified for 24 h with 2% nitric acid to determine an MDL. Ten replicate samples were run, and ^89^Y ICP-MS responses were measured. The average response was 2.12 ± 0.29 µg L^−1^ with a variance of 0.0849 and a calculated MDL of 0.8 µg L^−1^. Additional experiments were performed using a 5 µg L^−1^ MWCNT solution, which yielded an MDL of 0.49 µg L^−1^. As shown below, there was no Y detected in the supernatant of control experiments with wastewater biomass (i.e., absence of MWCNT). The ICP-MS method reduced the time required to analyze MWCNTs compared to spICP-MS because the spICP-MS used in this study requires additional external calculations outside of the instrument software to separate real pulses from particles versus baseline pulses. For spICP-MS, additional work is needed to calculate the efficiency of particle transport that reaches the detector. This requires purchasing and injecting particles of known size and concentration. As a result, we used ICP-MS rather than spICP-MS to analyze MWCNT removal by RAS (described below) by quantifying dissolved Y remaining in supernatant. There was no need to use spICP-MS in a quantitative manner. However, selected split samples analyzed using spICP-MS drew the same MWCNT removal conclusions as conventional ICP-MS analysis using Y, providing another indicator that Y is associated with MWCNTs.

### 3.4. Removal Efficiency of MWCNTs by Return Activated Sludge

**Figure 6:**
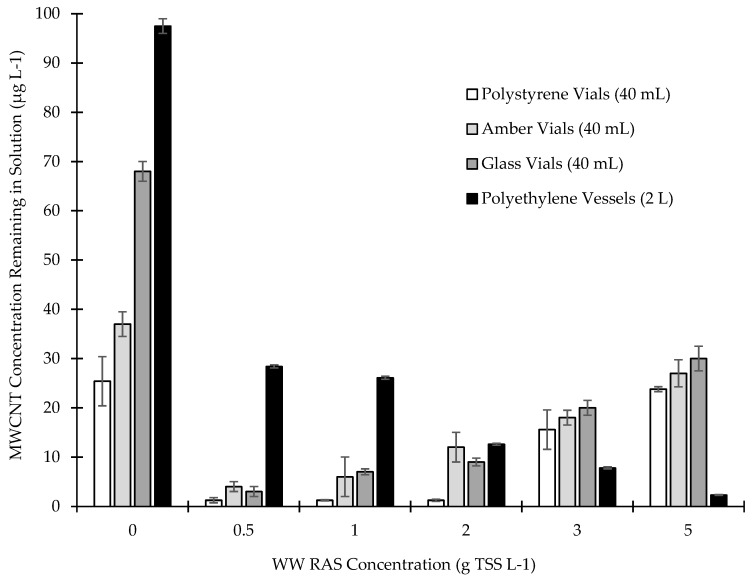
Effect of different vessels on MWCNTs removal by return activated sludge.

Figure 6 presents MWCNT removal by RAS in 40 mL polystyrene centrifuge vials, 40 mL glass vials, 40 mL amber vials, and 2 L polyethylene vials. Background Y concentrations in RAS were below the detection limit of ICP-MS. For the polystyrene centrifuge vials, only 25% of the MWCNTs spiked into the control samples (MWCNTs were added without RAS present) were detected in the supernatant by ICP-MS. The MWCNT loss was attributed to sorption on the polystyrene vial wall. In the experimental samples with low RAS biomass concentrations and in polystyrene vials, there was a further decrease in MWCNTs from the supernatant. However, at RAS concentrations above 3 g L^−1^, we observed higher concentrations of MWCNTs in the supernatant.

The experiments were repeated using glass vials and amber glass vials (Figure 6). There appeared to be less adsorption of MWCNTs to the vial wall in the control samples (no RAS) for both the amber vials and the glass vials compared with the polystyrene vials. The amber vials had ~36% MWCNTs remaining in solution without biomass. The glass vials had ~70% MWCNTs remaining in solution without biomass. Similar to polystyrene vials, at RAS concentrations above 3 g L^−1^, we observed higher concentrations of MWCNTs in the supernatant for both amber and glass vials.

We suspected that any surface may exhibit an affinity for a fixed mass of MWCNTs. Therefore, we decreased the reactor surface area to volume ratio by using 2 L polyethylene reactor vessels (0.85 cm^2^/cm^3^) instead of 40 mL vials (3.06 cm^2^/cm^3^). All samples were analyzed for dissolved Y, and a subset of samples were also analyzed by spICP-MS to confirm the presence/absence of MWCNTs in samples. Our control sample (no RAS) showed negligible loss of MWCNTs to vessel walls as >95% of MWCNTs spiked into 2 L vessel remained in the solution.

The RAS reduced the MWCNT concentrations from 100 µg L^−1^ to between 27 µg L^−1^ and 2.2 µg L^−1^ in the 2 L polyethylene reactor vessels (Figure 6). This indicates that as the RAS concentration increased, we achieved near complete MWCNT removal (97.7% MWCNT removal at 5 g TSS L^−1^ RAS). Based on triplicate experiments followed by Y analysis using ICP-MS and ANOVA (>95% confidence), there was a high reproducibility of the tests. These results showed that MWCNTs were well removed by wastewater RAS, showing consistency with previously published findings [13].

### 3.5. Implications of Using Yttrium as a Trace Residual Metal to Quantify MWCNT Removal by Wastewater Biomass

After completing WWTP RAS batch scale experiments, we considered a manufacturing spill scenario to understand how the RAS removal test strategy employed here could aid industry in meeting potential regulatory requirements. Obtaining EPA approval for use of nanomaterials may require estimating concentrations of MWCNTs in rivers receiving treated wastewater effluent from facilities impacted by industrial discharges. We considered a relatively small community of 20,000 residents. Assuming an average of 50 gallons per capita of sewage production, this equates to a 1 million gallons per day (MGD) WWTP. This is a common lower design capacity of activated sludge WWTPs. The scenario considered one 55-gallon drum of a 5% MWCNT solution leaked or spilled before being discharged to the sewer system over 24 h. In this scenario, the average MWCNT concentration entering the WWTP would be roughly 100–150 µg L^−1^.

We considered a manufacturing spill scenario to understand how the RAS removal test strategy employed here could aid industry in meeting potential regulatory requirements. Based upon results in Figure 6, an activated sludge WWTP operating at a biomass concentration of 1 to 3 g TSS L^−1^ would adsorb >95% of the MWCNTs and would result in 2–30 µg L^−1^ being released to receiving waters. MWCNTs at 2–30 µg L^−1^ would be below quantification limits by UV-Vis, Raman, PTA, etc. (Figures S1 and S2). Because Y concentrations in wastewater are <1 µg L^−1^ [26] the measurement of Y by ICP-MS or spICP-MS may be viable. Reported aquatic toxicity of MWCNTs are >50 µg L^−1^, and thus even without in-stream dilution, the spill event would be below this potential regulatory limit, even though the MWCNT concentration was >100 µg L^−1^ entering the WWTP in the raw sewage.

## 4. Conclusions

Detection and quantification of known commercially available MWCNTs at environmentally relevant concentrations (<50 µg L^−1^) using residual Y was found to be possible using both conventional ICP-MS and spICP-MS. Additionally, our detection limit of 2.5 µg L^−1^ was lower than many reported MWCNT concentrations in surface waters [36,37]. TEM imaging and dissolution studies showed that metal catalysts were physically bound to the MWCNT in bundles, indicating that they can be used as proxies for MWCNT quantification. In the net removal experiments, we observed that adding wastewater RAS at 500 mg TSS L^−1^ to 5 g TSS L^−1^ to solutions containing 100 µg L^−1^ MWCNT removed the MWCNT, with the RAS concentration at 5g TSS L^−1^ removing MWCNTs to below threshold reporting levels. This method is only applicable in lab experiments with known MWCNT, where Y content is a known constant. 

While Y was chosen as the surrogate trace residual metal in this study, each MWCNT will have distinctive synthesis methods [26,38,39] and thus will likely have unique trace residual metal compositions. Consequently, other researchers used Y or different trace elements to track CNTs (e.g., Co, Ni, Mo) [15,16,17,18,19,20,21,22]. It will be important to understand these differences between MWCNTs and whether a unified method can be developed to quantify MWCNTs through spICP-MS or ICP-MS using trace residual metals or whether trace residual metals like Y can be useful as a marker in standardized testing of MWCNTs moving forward. Y may not be appropriate for every MWCNT because each manufacturing process and source of catalyst materials (e.g., iron) may have unique trace element impurities. However, the methodology outlined herein, using multiple trace element selection criteria, should be applied for other applications of MWCNT detection at WWTPs.

## Figures and Tables

**Table 1:** Contents of trace metal residual elements in multi-walled carbon nanotubes (MWCNTs) determined by ICP-MS.

Metal	Concentration (μg gMWCNT^−1^)	* Sensitivity Regression Equation	* Sensitivity R^2^ Value
Fe	9363 ± 578	-	-
Y	76.19 ± 5.93	19,000 X + 1100	0.99
Zr	1253 ± 75.31	10,000 X + 2400	0.99
Co	153.3 ± 6.47	6200 X + 2600	0.99
Zn	10.47 ± 5.32	91 X + 970	0.78
Ni	5.06 ± 0.41	970 X + 182	0.99
Mo	1.19 ± 0.07	270 X + 4	0.99

Concentration data expressed as Mean ± SD of three independent measurements (µg g^−1^) * Data obtained from Figure 1.

## Supplementary Materials

The following figures are available online at https://www.mdpi.com/2079-4991/9/5/670/s1: Figure S1: Light scattering detection of MWCNTs, Figure S2: Programmed thermal analysis of MWCNTs, Figure S3: Calibration Curve for ^89^Y pulse counts (spICP-MS) versus dissolved Y concentrations (ICP-MS), Figure S4: Yttrium detection of standards and calibration curve using MWCNTs solution on spICP-MS, Figure S5: TEM images of 5% commercial MWCNTs solution, Figure S6: Detection limit calculations for ^89^Y by spICP-MS for MWCNTs.

## References

[1] Mohammadzadeh F., Jahanshahi M., Rashidi A. (2012). Preparation of nanosensors based on organic functionalized MWCNT for H_2_S detection. Appl. Surf. Sci..

[2] Dorraji S., Ahadzadeh I., Rasoulifard M. (2014). Chitosan/polyaniline/ MWCNT nanocomposite fibers as an electrode material for electrical double layer capacitors. Int. J. Hydrog. Energy.

[3] Asmatulu R., Mahmud G.A., Hille C., Misak H.E. (2011). Effects of UV degradation on surface hydrophobicity, crack, and thickness of MWCNT-based nanocomposite coatings. Prog. Org. Coat..

[4] Kuznetzov A.A., Lee S.B., Zhang M., Baughman R.H., Zakhidov A. (2010). Electron field emission from transparent multiwalled carbon nanotube sheets for inverted field emission displays. Carbon.

[5] Mueller N., Nowack B. (2008). Exposure modelling of engineered nanoparticles in the environment. Environ. Sci. Technol..

[6] Gottschalk F., Sonderer T., Scholz R., Nowack B. (2009). Modeled environmental concentrations of engineered nanomaterials (TiO_2_, ZnO, Ag, CNT, Fullerenes) for different regions. Environ. Sci. Technol..

[7] Petersen E., Zhang L., Mattison N., O’Carroll D., Whelton A., Uddin N., Nguyen T., Huang Q., Henry T., Holbrook D. (2011). Potential Release Pathways, Environmental Fate, and Ecological Risks of Carbon Nanotubes. Environ. Sci. Technol..

[8] Garner K., Suh S., Lenihan H., Keller A. (2015). Species sensitivity distributions for engineered nanomaterials. Environ. Sci. Technol..

[9] Jackson P., Jacobsen N., Baun A., Birkedal R., Kuhnel D., Jensen K., Vogel U., Wallin H. (2013). Biaccumulation and ecotoxicity of carbon nanotubes. Chem. Cent. J..

[10] Kiser M.A., Ladner D.A., Hristovski K.D., Westerhoff P. (2012). Nanomaterial transformation and association with fresh and freeze-dried wastewater activated sludge: Implications for testing protocol and environmental fate. Environ. Sci. Technol..

[11] Kiser M.A., Westerhoff P., Benn T., Wang Y., Pérez-Rivera J., Hristovski K. (2009). Titanium nanomaterial removal and release from wastewater treatment plants. Environ. Sci. Technol..

[12] Westerhoff P.K., Kiser M.A., Hristovski K. (2013). Nanomaterial removal and transformation during biological wastewater treatment. Environ. Eng. Sci..

[13] Yang Y., Yu Z., Nosaka T., Doudrick K., Hristovski K., Herckes P., Westerhoff P. (2015). Interaction of carbonaceous nanomaterials with wastewater biomass. Front. Environ. Sci. Eng..

[14] Petersen E., Cervantes D., Bucheli T., Elliott L., Fagan J., Gogos A., Hanna S., Mansfield E.K.R., Bustos A., Plata D. (2016). Quantification of carbon nanotubes in environmental matrices: Current capabilities, cases studies and future prospects. Environ. Sci. Technol..

[15] Wang J., Lankone R., Reed R., Fairbrother H., Ranville J. (2016). Analysis of single-walled carbon nanotubes using spICP-MS with microsecond dwell time. NanoImpact.

[16] Rasmussen P.E., Avramescu M., Jawawardene I., Gardner D. (2015). Detection of Carbon Nanotubes in Indoor Workplaces Using Elemental Impurities. Environ. Sci. Technol..

[17] Lim J.H., Bairi V.G., Fong A. (2017). Quantification of impurities in carbon nanotubes: Development of ICP-MS sample preparation methods. Mater. Chem. Phys..

[18] Goodwin D.G., Adeleye A., Sung L., Ho K., Burgess R., Petersen E. (2018). Detection and Quantification of Graphene-Family Nanomaterials in the Environment. Environ. Sci. Technol..

[19] Avramescu M.L., Rasmussen P.E., Chenier M. (2016). Determination of Metal Impurities in Carbon Nanotubes Sampled Using Surface Wipes. J. Anal. Methods Chem..

[20] Wohlleben W., Kingston C., Carter J., Sahle-emessie E., Vazquez-Campos S., Acrey B., Chen C., Walton E., Egenolf H., Muller P. (2017). NanoRelease: Pilot interlaboratory comparison of a weathering protocol applied to resilient and labile polymers with and without embedded carbon nanotubes. Carbon.

[21] Tromp P.C., Kuijpers E., Bekker C., Godderis L., Lan Q., Jedynska A.D., Vermeulen R., Pronk A. (2017). A New Approach Combining Analytical Methods for Workplace Exposure Assessment of Inhalable Multi-Walled Carbon Nanotubes. Ann. Work Expo. Health.

[22] Kato N., Nagaya T., Matsui Y., Yoneda M. (2017). Exposure assessment of carbon nanotubes at pilot factory focusing on quantitative determination of catalytic metals. J. Occup. Health.

[23] Reed R.B., Goodwin D.G., Marsh K.L., Capracotta S.S., Higgins C.P., Fairbrother D.H., Ranville J.F. (2013). Detection of single walled carbon nanotubes by monitoring embedded metals. Environ. Sci. Process. Impacts.

[24] Montano M.D., Badiei H.R., Bazargan S., Ranville J. (2014). Improvements in the detection and characterization of engineered nanoparticles using spICP-MS with microsecond dwell times. Environ. Sci. Nano.

[25] Kiser M.A., Ryu H., Jang H., Hristovski K., Westerhoff P. (2010). Biosorption of nanoparticles to heterotrophic wastewater biomass. Water Res..

[26] Doudrick K., Herckes P., Westerhoff P. (2012). Detection of carbon nanotubes in environmental matrices using programmed thermal analysis. Environ. Sci. Technol..

[27] Corredor C., Hou W., Klein S., Moghadam B., Goryll M., Doudrick K., Westerhoff P., Posner J. (2013). Disruption of model cell membranes by carbon nanotubes. Carbon.

[28] Silva R.M., Doudrick K., Franzi L.M., TeeSy C., Anderson D.S., Wu Z., Pinkerton K.E. (2014). Instillation versus inhalation of multiwalled carbon nanotubes: Exposure-related health effects, clearance, and the role of particle characteristics. ACS Nano.

[29] Doudrick K., Nosaka T., Herckes P., Westerhoff P. (2015). Quantification of graphene and graphene oxide in complex organic matrices. Environ. Sci. Nano.

[30] Ge C., Lao F., Li W., Li Y., Chen C., Qiu Y., Mao X., Li B., Chai Z., Zhao Y. (2008). Quantitative Analysis of Metal Impurities in Carbon Nanotubes: Efficacy of Different Pretreatment Protocols for ICPMS Spectroscopy. Anal. Chem..

[31] Lee S., Bi X., Reed R., Ranville J., Herckes P., Westerhoff P. (2014). Nanoparticle size detection limits by single particle ICP-MS for 40 elements. Environ. Sci. Technol..

[32] Bi X., Lee S., Ranville J.F., Sattigeri P., Spanias A., Herckes P., Westerhoff P. (2014). Quantitative resolution of nanoparticle sizes using single particle inductively coupled plasma mass spectrometry with the K-means clustering algorithm. J. Anal. Spectrom..

[33] Mitrano D.M., Ranville J.F., Bednar A., Kazor K., Hering A.S., Higgins C.P. (2014). Tracking dissolution of silver nanoparticles at environmentally relevant concentrations in laboratory, natural, and processed waters using single particle ICP-MS (spICP-MS). Environ. Sci. Nano.

[34] Westerhoff P., Lee S., Yang Y., Gordon G., Hristovski K., Halden R., Herckes P. (2015). Characterization, recovery opportunities, and valuation of metals in municipal sludges from US wastewater treatment plants nationwide. Environ. Sci. Technol..

[35] Pace H., Rogers N., Jarolimek C., Coleman V., Higgins C., Ranville J. (2011). Determining transport efficiency for the purpose of counting and sizing nanoparticles via single particle inductively coupled plasma mass spectrometry. Anal. Chem..

[36] Bouchard D., Knightes C., Chang X., Avant B. (2017). Simulating multiwalled carbon nanotube transport in surface water systems using the water quality analysis simulation program (wasp). Environ. Sci. Technol..

[37] Gottschalk F., Nowack B. (2011). The release of engineered nanomaterials to the environment. J. Environ. Monit..

[38] Eatemadi A., Daraee H., Karimkhanloo H., Kouhi M., Zarghami N., Akbarzadeh A., Abasi M., Hanifehpour Y., Joo S. (2014). Carbon nanotubes: Properties, synthesis, purification, and medical applications. Nanoscale Res. Lett..

[39] Kateb B., Yamamoto V., Alizadeh D., Zhang L., Manohara H., Bronikowski M., Badie B. (2010). Multi-walled carbon nanotube (MWCNT) synthesis, preparation, labeling, and functionalization. Methods Mol. Biol..

