# Supplementary material for: Yttrium Residues in MWCNT Enable Assessment of MWCNT Removal during Wastewater Treatment
Source: Nanomaterials (Basel). 2019 May 1;9(5):670. doi: 10.3390/nano9050670 (PMC6566316; doi:10.3390/nano9050670)
Supplement: Supplementary file 1 [file nanomaterials-09-00670-s001.pdf]

Supplemental Information (SI):

1. *Light Scattering Detection of MWCNTs*

UV-Vis light scattering was first investigated to detect MWCNTs in solution. MWCNTs exhibit a broad response between 300 and 800 nm wavelengths (Figure S1A). Calibration curves were generated for both 300 nm and 400 nm wavelengths (Figure S1B) and were linear between 1 and 10 mg $L^{-1}$. Based upon the absorbance of MWCNTs and background absorbance of treated wastewater, the UV-Vis method for MWCNT determination was deemed valid only to ~ 0.5 mg $L^{-1}$ of MWCNT.

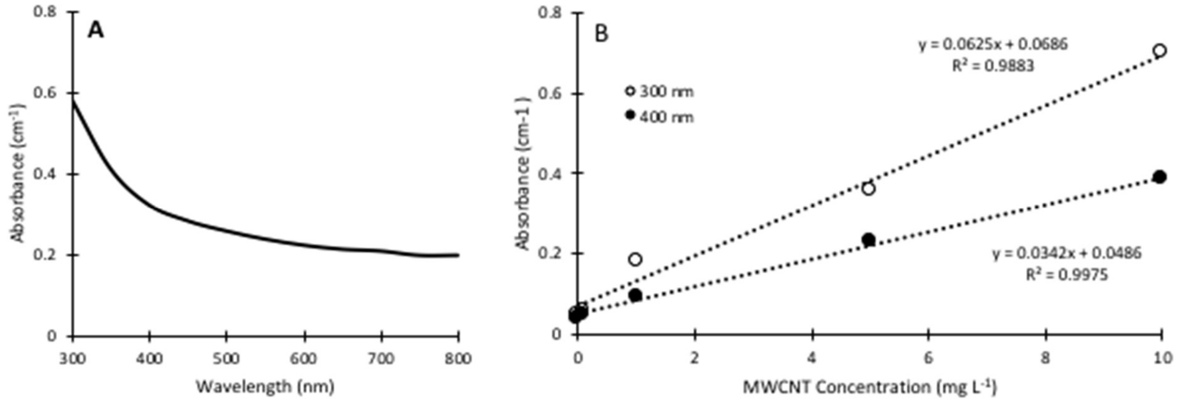


**Figure S1**. The left figure (A) shows the UV-Vis spectra for 10 mg $L^{-1}$ MWCNT on a 300–800 nm wavelength scan. There is no optimal peak, so calibration curves in the right figure (B) were made for MWCNT at 300 nm and 400 nm wavelengths. These are commonly used wavelengths for carbon nanotubes

## 2. *Programmed Thermal Analysis of MWCNTs*

PTA was also attempted to quantify MWCNTs in water. Figure S2 shows PTA temperature profile applied (black line) and two PTA thermograms with a flame ionization detector (FID) carbon signal response (y-axis) for a dry MWCNT and then the material dried from the 5% MWCNT solution. The dry (powder) MWCNT product (red line) had a low response in the inert gas range (0–450 seconds) and then two peaks in the presence of oxygen gas (450–1000 seconds), which is typical of relatively pure CNTs with low organic content. The PTA thermogram for the MWCNT 5% solution (blue line) had a large response in the inert gas region, which is typical when organic compounds are present, and then a response that is shifted towards higher temperatures during exposure to the oxygen gas environment. The high background organic content from the surfactant used to stabilize the MWCNTs prevented the use of PTA for quantifying MWCNTs in water.

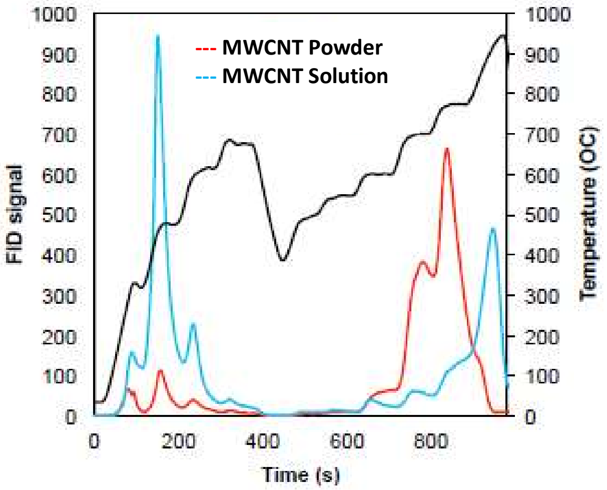


**Figure S2**. PTA temperature profile (black line) and FID signal response thermograms for two MWCNT products, one dry (red-line) and one liquid (blue-line).

3. *Calibration Curve for $^{89}$Y Pulse Counts (spICP-MS) Versus Dissolved Y Concentrations (ICP-MS)*

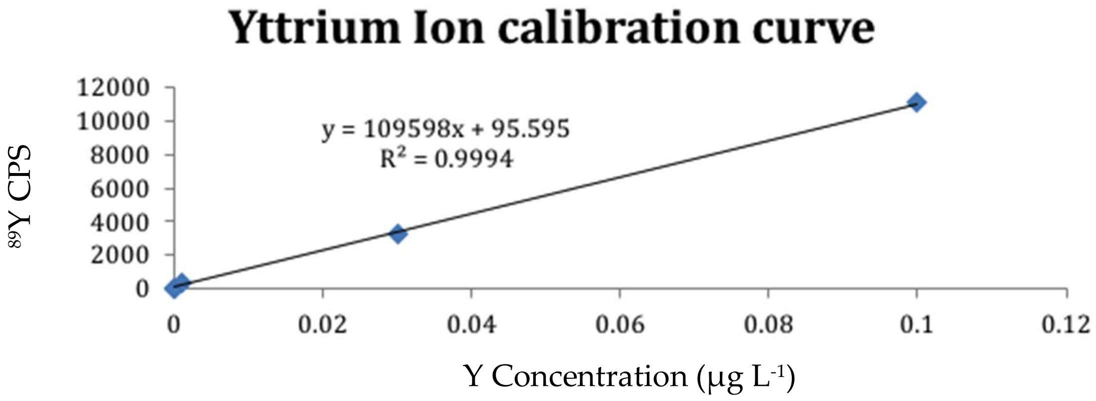


**Figure S3**. Calibration Curve for $^{89}$Y pulse counts (spICP-MS) versus dissolved Y concentrations (ICP-MS). This calibration curve was used to determine MWCNTs remaining in solution with ICP-MS. The 5% MWCNT solution was diluted to between 0.001 and 0.10 μg $L^{-1}$.

4. *Calibration Curve for yttrium pulse counts versus MWCNT concentration using ICP-MS*

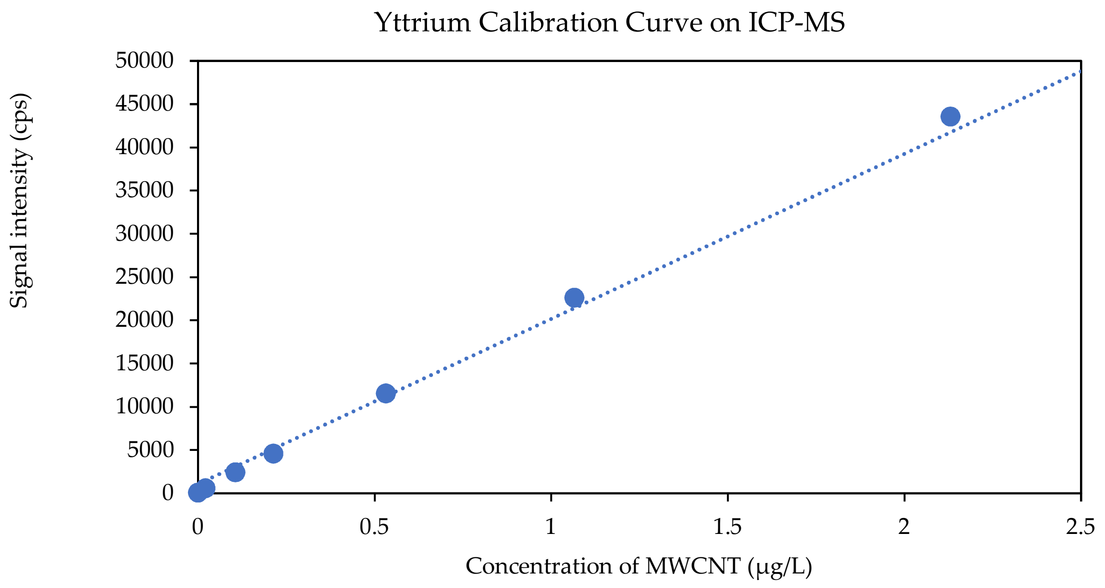


Figure S4. Calibration Curve for yttrium pulse counts versus MWCNT concentration using ICP-MS

5. *TEM Images of MWCNT Solution*

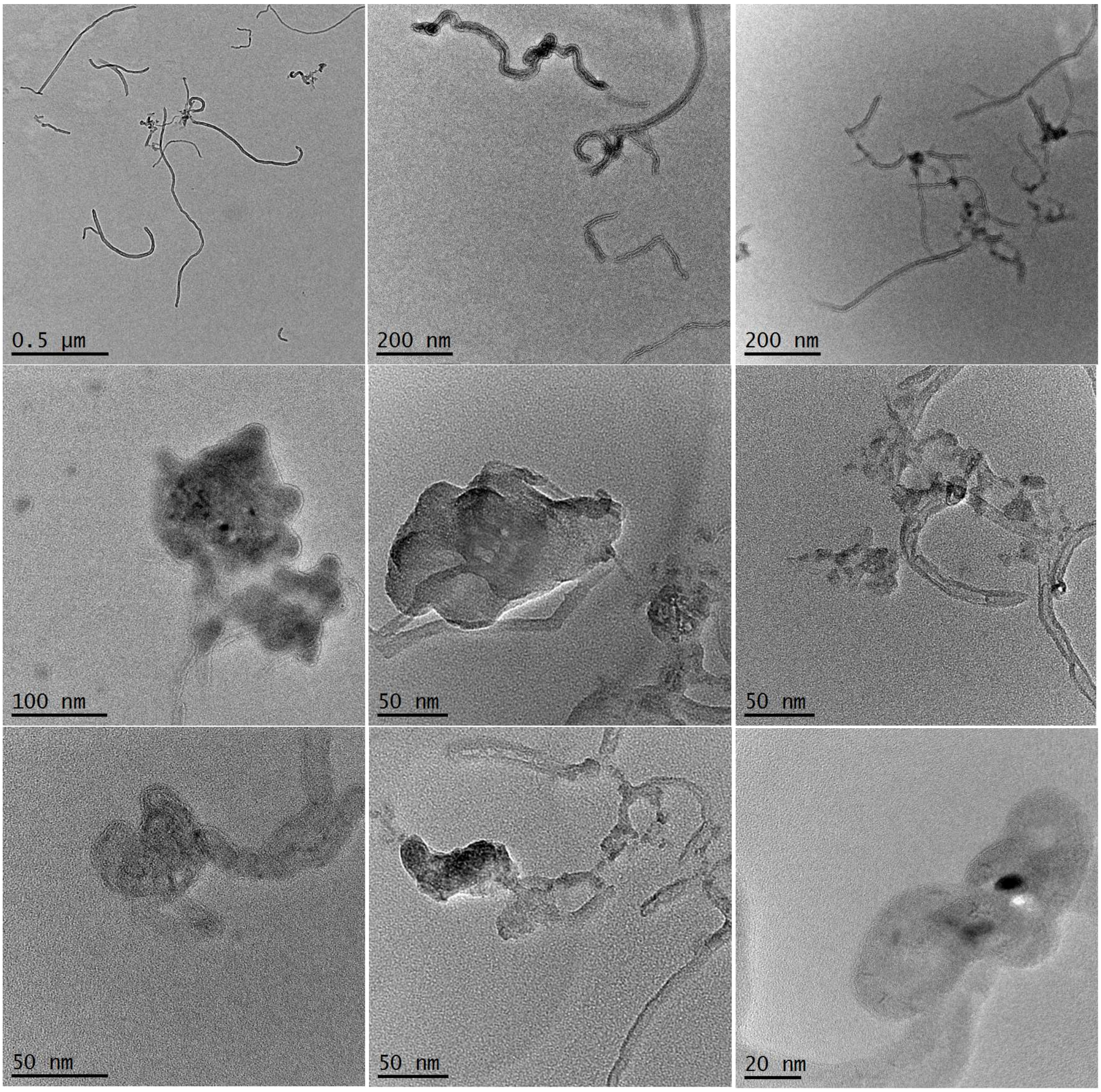


**Figure S5**. TEM images of MWCNTs in 1 mM $NaHCNO_3$ matrix solution.

6. *Detection Limit Calculations for $^{89}Y$ by spICP-MS for MWCNTs*

Y by ICP for MWCNT

Test concentration = 2 ug/L

| Sample # | C (ppb) | |
|---|---|---|
| 1 | 2.199 | 0.0022 |
| 2 | 2.225 | 0.00222 |
| 3 | 2.402 | 0.0024 |
| 4 | 2.403 | 0.0024 |
| 5 | 2.239 | 0.00224 |
| 6 | 1.856 | 0.00186 |
| 7 | 1.859 | 0.00186 |
| 8 | 1.758 | 0.00176 |
| 9 | 1.744 | 0.00174 |
| 10 | 2.524 | 0.00252 |

| | |
|---|---|
| n | 10 |
| Average | 2.12075 |
| Variance | 0.08487 |
| StDev | 0.29133 |
| t (n-1, 0.01) | 2.821 |

t value for 99% at n (from Table)

MDL 0.822

t value * StDev

| | $X^2/df$ | $(X^2/df)$ (MDL) |
|---|---|---|
| LCL | 0.300 | 0.247 |
| UCL | 2.114 | 1.737 |

Test concentration = 5 ug/L

| Sample # | C (ppb) |
|---|---|
| 1 | 4.869 |
| 2 | 5.079 |
| 3 | 5.143 |
| 4 | 5.259 |
| 5 | 4.993 |
| 6 | 4.958 |
| 7 | 5.119 |
| 8 | 5.296 |
| 9 | 4.794 |
| 10 | 4.834 |

| | |
|---|---|
| n | 10 |
| Average | 5.03428 |
| Variance | 0.03031 |
| StDev | 0.17411 |
| t (n-1, 0.01) | 2.821 |

t value for 99% at n (from Table)

MDL 0.491

t value * StDev

| | $X^2/df$ | $(X^2/df)$ (MDL) |
|---|---|---|
| LCL | 0.300 | 0.147 |
| UCL | 2.114 | 1.038 |

| Var ratio | F-ratio |
|---|---|
| 0.357177 | 5.35 |

from table

if $S^2_A/S^2_B < 5.35$, then compute the pooled standard deviation

S pooled 0.23999

MDL 0.612

| | $X^2/df$ | $(X^2/df)$ (MDL) |
|---|---|---|
| LCL | 0.469 | 0.230 |
| UCL | 1.729 | 0.849 |

t table

| df | t |
|---|---|
| 6 | 3.143 |
| 7 | 2.998 |
| 8 | 2.896 |
| 9 | 2.821 |
| 10 | 2.764 |
| 11 | 2.718 |
| 12 | 2.681 |
| 13 | 2.650 |
| 14 | 2.624 |
| 15 | 2.602 |
| 16 | 2.583 |
| 17 | 2.567 |
| 18 | 2.552 |
| 19 | 2.539 |
| 20 | 2.528 |
| 21 | 2.518 |
| 22 | 2.508 |
| 23 | 2.500 |
| 24 | 2.492 |
| 25 | 2.485 |
| 26 | 2.479 |
| 27 | 2.473 |
| 28 | 2.467 |
| 29 | 2.462 |
| 30 | 2.457 |

**Figure S6.** Raw data for calculating minimum detection limits of MWCNT 5% solution using $^{89}Y$ measurements on spICP-MS.
